# Composite Hydrogels with Embedded Silver Nanoparticles and Ibuprofen as Wound Dressing

**DOI:** 10.3390/gels9080654

**Published:** 2023-08-14

**Authors:** Irina Popescu, Marieta Constantin, Gheorghe Solcan, Daniela Luminita Ichim, Delia Mihaela Rata, Loredana Horodincu, Carmen Solcan

**Affiliations:** 1 “Petru Poni” Institute of Macromolecular Chemistry, Grigore Ghica Voda Alley 41A, 700487 Iasi, Romania; ipopescu@icmpp.ro (I.P.); marieta@icmpp.ro (M.C.); 2 Faculty of Veterinary Medicine, “Ion Ionescu de la Brad” Iasi University of Life Sciences, 700489 Iasi, Romania; gsolcan@uaiasi.ro (G.S.); loredana.horodincu@uaiasi.ro (L.H.); 3 Faculty of Medical Dentistry, “Apollonia” University of Iasi, 700511 Iasi, Romania; danielaluminitaichim@yahoo.com (D.L.I.); delia.rata@univapollonia.ro (D.M.R.)

**Keywords:** chitosan/PVA hydrogels, silver nanoparticles, ibuprofen, antibacterial activity, wound healing

## Abstract

The wound healing process is often slowed down as a result of complications from bacterial infections and inflammatory reactions. Therefore, it is necessary to develop dressings with fast antibacterial and anti-inflammatory activity that shorten the wound healing period by promoting cell migration and proliferation. Chitosan (CS)-based hydrogels have been widely studied for their antibacterial and wound healing capabilities. Herein, we developed a composite hydrogel based on CS and PVA embedding silver nanoparticles (AgNPs) with antibacterial properties and ibuprofen (Ib) as an anti-inflammatory agent. The hydrogel prepared by double physical cross-linking, with oxalic acid and by freeze–thawing, loaded with 0.225 wt.% AgNPs and 0.264 wt.% Ib, displayed good mechanical properties (compressive modulus = 132 kPa), a high swelling degree and sustained drug delivery (in simulated skin conditions). Moreover, the hydrogel showed strong antibacterial activity against *S. aureus* and *K. pneumoniae* due to the embedded AgNPs. In vivo, this hydrogel accelerated the wound regeneration process through the enhanced expression of TNF alpha IP8, by activating downstream cascades and supporting the healing process of inflammation; Cox2, which enhances the migration and proliferation of cells involved in re-epithelization and angiogenesis; MHCII, which promotes immune cooperation between local cells, eliminating dead tissue and controlling infection; the intense expression of Col I as a major marker in the tissue granulation process; and αSMA, which marks the presence of myofibroblasts involved in wound closure and indicates ongoing re-epithelization. The results reveal the potential healing effect of CS/PVA/AgNPs/Ib hydrogels and suggest their potential use as wound dressings.

## 1. Introduction

The field of wound management poses challenges to both clinicians and researchers within various aspects of medicine. Recent advancements in this area involve incorporating innovative technologies that target wounds at a cellular level, moving beyond mere moisture reduction. Successful wound healing involves the complex orchestration of biological and molecular events, including cell migration, cell proliferation and extracellular matrix deposition. For cutaneous injuries that heal without underlying pathophysiological issues (acute wounds), the body’s primary goal is rapid repair with minimal energy expenditure. Consequently, such wounds typically heal with scars and lack regeneration [1]. However, wounds with underlying pathophysiological abnormalities, such as venous leg ulcers, diabetic foot ulcers and full-thickness burn injuries, experience impaired healing due to a lack of evolutionary adaptations. The process of wound healing comprises several interconnected elements, mainly inferred from experimental animal wounds. These elements cannot be distinctly separated and classified. Instead, it is helpful to understand the repair process as four overlapping phases: coagulation, inflammation, proliferation (including matrix deposition) and remodeling [1]. Each phase is influenced by different cytokines and growth factors. Inflammation has a positive role in the healing process, but a disproportionate or prolonged inflammatory response is undesirable because it interferes with the other stages of the healing process, causing an increase in the wound size and excessive scarring. Different strategies can be used to prevent an acute inflammatory phase, including the use of non-steroidal anti-inflammatory drugs (NSAID) or phyto-modulators [2]. Modern interactive dressings play a vital role in facilitating wound healing. They actively modify the wound’s environment and interact with its surface to optimize the healing process. These dressings possess the capability to work effectively with wound components such as exudate, tissues, cells and growth factors to enhance the healing process. However, selecting the most appropriate dressing for a particular wound can be challenging. Successful wound management requires a deep understanding of the tissue repair process and knowledge about the available properties of dressings [3].

Hydrogels obtained by the chemical or covalent cross-linking of polymers are known for their applications as wound dressing materials due to their biocompatibility, non-toxicity and high water swelling capacity, which allow the absorption of exudate, as well as gas permeability and good mechanical properties [4]. Their ability to act as controlled drug delivery systems, or the per se bioactivity of the hydrogel components, represents an advantage in the wound healing process [5]. Natural polymers such as chitosan, alginate and collagen are the most frequently used in wound dressing and tissue engineering; however, synthetic polymers, even if they do not actively participate in the wound healing process, are used in combination with natural polymers to obtain the desired properties of the material [5,6]. Chitosan (CS), a biocompatible cationic polysaccharide, is known for its hemostatic, anti-inflammatory and antimicrobial activity and its important role in skin wound healing [7,8]. On the other hand, polyvinyl alcohol (PVA) is known to be used in wound dressing, especially in combination with polysaccharides to improve the physical properties of the material [9]. Physically cross-linked PVA hydrogels obtained by the freeze–thawing procedure are candidates for diverse biomedical applications due to their gelation under mild conditions, lack of low molecular impurities, high porosity and good mechanical strength [10]. The incorporation of CS in a high amount into PVA freeze–thawed hydrogels leads to the formation of materials with a non-regular structure, weak mechanical properties and low stability in aqueous environments [11,12]. This is why additional cross-linking of the polysaccharide is required to obtain PVA/CS hydrogels with increased content of CS [13]. In a previous paper, we proved that oxalic acid can be used as an excellent cross-linker to improve the gel fraction and the mechanical properties of PVA/CS hydrogels [14]. The lack of toxic cross-linkers and the tailored porosity of these hydrogels represent the first indications of their potential use as wound dressing materials. Metallic nanoparticles (silver, gold, zinc oxide, copper), known for their biological activity, can be used as adjuvants in the treatment of wounds, reducing infections and accelerating the healing process [15,16]. Due to their low cost and chemical stability, but especially to their high antibacterial activity with great efficiency toward drug-resistant pathogens from chronic wounds, silver nanoparticles (AgNPs) are considered good candidates in this field [17]. Their incorporation into polymeric matrices leads to the obtaining of new composite materials, extensively studied due to their biomedical applications [18,19,20,21,22]. Apart from the antimicrobial activity of a wound dressing, its ability to deliver drugs capable of preventing excess inflammation and reducing pain would represent an advantage. Ibuprofen, a non-steroidal anti-inflammatory drug, is often used in medicines due to its analgesic and antipyretic effects, being well tolerated. The anti-inflammatory effect of ibuprofen arises from its interaction with the cyclooxygenase-2 enzyme [23]. Although NSAIDs can have a negative effect on wound healing, their short-term use in the first stage affects the cytokine response, decreases inflammation and accelerates healing [24]. Thus, it was demonstrated that dressings containing ibuprofen improve skin wound healing [25,26]. Despite its anti-inflammatory potency, the limitations of ibuprofen use in the therapy of skin lesions are due to its low solubility, short biological half-life and rapid clearance after oral administration [27,28,29]. To overcome these limitations and to enable long-term and sustained drug delivery, researchers have turned to the transdermal drug delivery system [30,31]. This system offers advantages such as the avoidance of first-path metabolism, stable and controlled blood levels, easy cessation of drug action, extended duration of effects and a lack of interference with gastric and intestinal fluids [32,33,34,35,36]. However, creating effective transdermal formulations for ibuprofen presents challenges because of the above. Nevertheless, there is significant interest in developing novel transdermal formulations to reduce dose wastage and side effects [37,38,39]. Researchers are actively investigating various techniques and materials to address these issues and improve the efficacy of ibuprofen delivery through the transdermal route. Potential approaches include the use of polymeric aerogels as promising materials for transdermal delivery systems [27,40,41]. The novelty of the present work results from the loading of the hydrogels with AgNPs as an antibacterial agent, in addition to ibuprofen as an anti-inflammatory drug, for application on the surface of damaged skin.

In a previous paper, we demonstrated that CS acts as a reducing and stabilizing agent in the synthesis of AgNPs. Covering the surface of the metallic nanoparticles, CS can be further involved in the physical cross-linking [14]. Thus, PVA/CS/AgNPs composite hydrogels can be obtained by double cross-linked procedures: freeze–thawing and the physical cross-linking of CS with oxalic acid. These methods allow the introduction of the desired amount of AgNPs uniformly distributed in the hydrogel. In the present work, PVA/CS/AgNPs composite hydrogels were first obtained and then loaded with ibuprofen. The hydrogels were characterized from the point of view of their composition, porosity, swelling, mechanical properties and drug release mechanism. The influence of the AgNPs and ibuprofen addition on the antibacterial activity of the new materials against *Staphylococcus aureus* and *Klebsiella pneumoniae* was investigated by the disc diffusion method.

Rabbits were frequently used many years ago as a model for wound healing. Wounds can be induced through various means, including biopsy, and may involve lesions affecting the epidermis, dermis and cartilage [42]. The healing process in this model occurs from the wound’s inner edges, and, unlike murine models, there is no shrinkage observed. A significant advantage of this model is that wounds heal through re-epithelization [43,44,45]. Moreover, it allows for the use of a small number of animals while still providing sufficient data for within-animal replications, with the possibility of creating up to six wounds [44]. In general, in vivo wound models offer several benefits. They enable the examination of interactions between multiple cell populations and body systems during the repair process. Additionally, these models allow for the investigation of various aspects of wound healing, including the selective reduction of specific genes to assess their impact on the process. They also provide opportunities to study the reactivity of the immune system during healing. Within a single animal, researchers can create multiple wounds, and the models can be adapted to study different wound healing patterns, such as burns, surgery-induced wounds, crush injuries and more [43,46,47].

Therefore, the aim of the present study was to demonstrate the wound dressing power of composite hydrogels with antibacterial properties containing a well-defined amount of AgNPs and ibuprofen. The aims of this work were (i) determining the direct interaction of the composite hydrogel with the wound through its physico-chemical properties; (ii) the use of composite hydrogels as antimicrobial agents in vitro; and (iii) the use of composite hydrogels as a stimulant for wound healing in vivo.

## 2. Results and Discussion

### 2.1. Preparation and Characterization of the Composite Hydrogels with and without Ibuprofen

**Figure 1:**
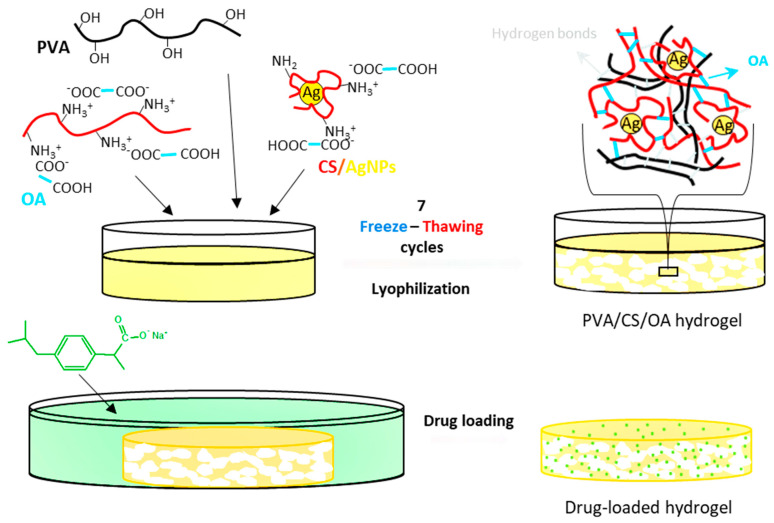
Preparation process of hydrogels containing AgNPs and ibuprofen.

In order to obtain composite hydrogels with appropriate properties (swelling ratio, porosity, mechanical strength) to be applied as materials for wound healing, hydrogels containing AgNPs and loaded with an anti-inflammatory drug were obtained by a method previously described by Popescu et al. [14]. The hydrogels were obtained by the freeze–thawing technique, a method that led to the formation of hydrogen bonds especially between PVA macromolecules, but also between CS chains, between CS and PVA and between oxalic acid and CS or PVA. In addition to the hydrogen bonds, electrostatic interactions between oxalic acid and chitosan took place in the hydrogels (Figure 1). On the other hand, the CS that generated and covered the AgNPs was also involved in the physical interactions, leading to the entrapment of the nanoparticles in the polymeric matrix. The free remaining amine groups from CS in the hydrogel were used in the electrostatic interactions with the anionic drug, ibuprofen. The presence of all these components in the hydrogels, as well as their interactions, were first demonstrated by FT-IR spectroscopy.

**Figure 2:**
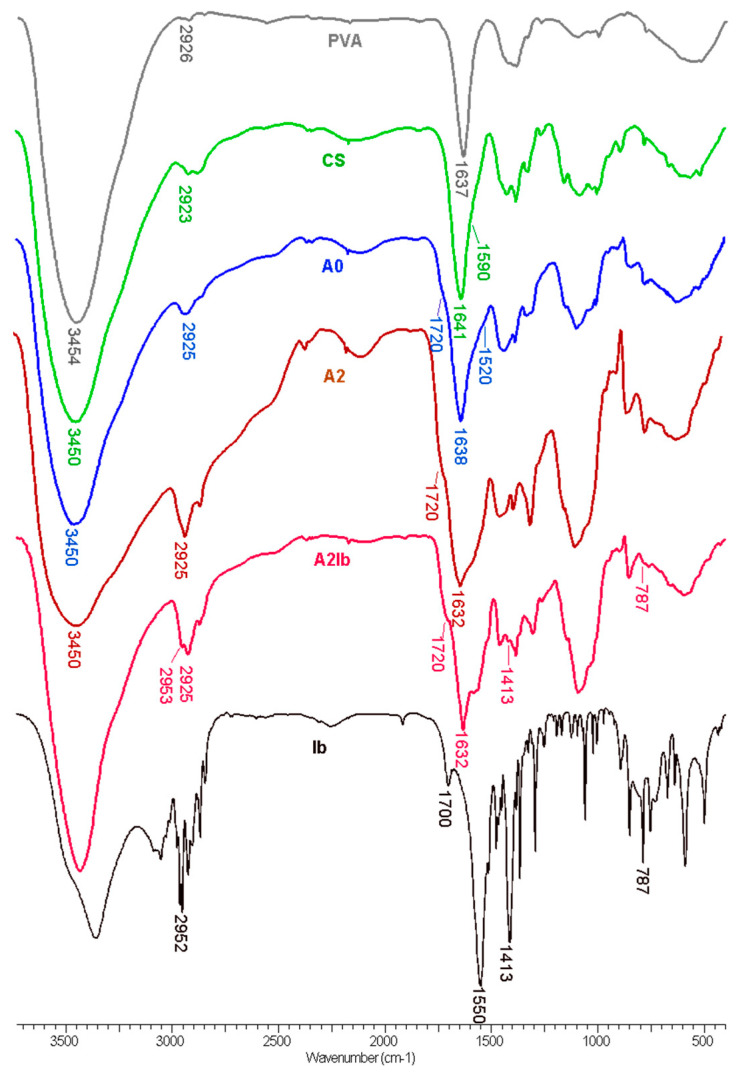
FT-IR spectra of PVA, CS, hydrogels without AgNPs (**A0**) and with embedded AgNPs (**A2**), hydrogel containing both AgNPs and ibuprofen (**A2Ib**) and ibuprofen sodium salt (**Ib**).

The FT-IR spectra of the hydrogels together with the parent polymers and the pure drug are presented in Figure 2. In the spectrum of PVA, we can observe the peaks related to the hydroxyl (3450 cm^−1^) and acetate groups left in the polymeric chains (1637 cm^−1^). The spectrum of CS presents the characteristic bands from 3460 cm^−1^ (-OH and -NH_2_ groups), 1641 cm^−1^ (amide I), and 1560 cm^−1^—shoulder (NH_2_ band). In the spectrum of the hydrogel **A0**, obtained from PVA, CS and oxalic acid, a shoulder appears at 1520 cm^−1^ due to the ionization of the amine groups of CS (-NH_3_^+^), and the amide band moves from 1641 in CS to 1638 cm^−1^ in the hydrogel due to the interaction with di-carboxylic acid [48,49]. The presence of oxalic acid is evidenced by the shoulder from 1720 cm^−1^ (COOH groups). A peak from 2925 cm^−1^, attributed to the methylene groups from PVA and from the pyranose ring of CS, is also found in the spectrum of the **A0** hydrogel.

When CS-AgNPs were introduced in the hydrogel—the **A2** sample—the amide I band was observed at 1632 cm^−1^, compared to 1638 cm^−1^ in the **A0** sample. This blue shift can be explained by the interaction between nitrogen atoms from amide groups and the silver nanoparticles [31]. When ibuprofen was loaded in the composite hydrogel (**A2Ib** sample), some of the characteristic peaks from the drug were observed: 2952 cm^−1^ (CH_3_ asymmetric stretching), 1413 cm^−1^ (CH-CO deformation), and 787 cm^−1^ (CH_2_ rocking) [50]. The bands from the carboxylic acid (1700 cm^−1^) and carboxylate ions (1550 cm^−1^) in sodium ibuprofen were overlapped by the bands of the hydrogel.

**Figure 3:**
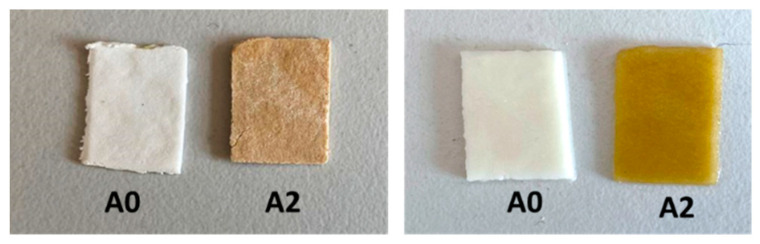
Optical images of **A0** and **A2** samples in dried (**left panel**) and swollen state (**right panel**).

The exact amount of CS and silver in the hydrogels was determined by a ninhydrin assay and AAS, respectively, and the results are presented in Table 1. The theoretical content of CS was 33 wt.% in the samples without the drug (**A0** and **A2**) and around 26 wt.% in the drug-loaded hydrogels (**A0Ib** and **A2Ib**). The values determined experimentally were higher than the theoretical ones due to the leakage of the excess of the oxalic acid and of the uncross-linked polymers during the purification step. The content of silver in the **A2** and **A2Ib** samples was slightly smaller compared to the theoretical composition (3.0 mg silver/g). The drug loading in the hydrogels was relatively high (260 mg/g), and the value for **A0Ib** was close to the value obtained for the **A2Ib** sample, as expected. The presence of the AgNPs in the purified hydrogels was also demonstrated by the brown-yellow color of the **A2** sample, compared with the **A0** sample, which was white (Figure 3).

**Figure 4:**
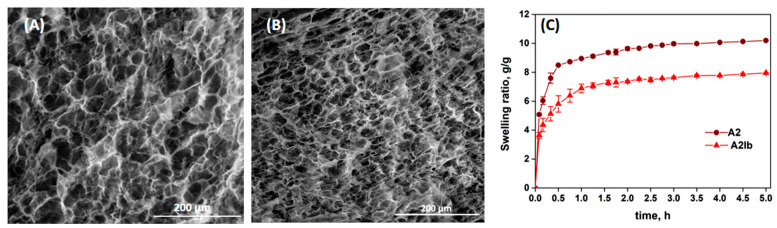
SEM images of **A2** (**A**) and **A2Ib** (**B**) hydrogels, and the swelling ratio of the hydrogels in buffer pH = 5.5 vs. time (**C**).

The morphology of the composite hydrogels before and after the absorption of ibuprofen was studied by SEM and the images are presented in Figure 4A,B. The hydrogel **A2** showed a porous structure with interconnected pores produced by the ice crystals usually obtained during the freeze–thawing process [51]. The absorption of the drug led to a decrease in the average diameter of pores from 27 µm in the **A2** sample to 22 µm in the **A2Ib** hydrogel, due to the folding/collapse of the CS chains after the association with ibuprofen.

The swelling kinetics of the hydrogels in PB with pH = 5.5 are presented in Figure 4C. Due to the presence of large pores, the solvent diffused easily and the hydrogels showed high rates of swelling in the first hour. The swelling capacity was lower for the drug-loaded hydrogel (**A2Ib**) compared with the un-loaded hydrogel. This can be explained by the lower porosity of the **A2Ib** sample (see Figure 4B) and probably by the hydrophobicity brought by the ibuprofen molecules.

**Figure 5:**
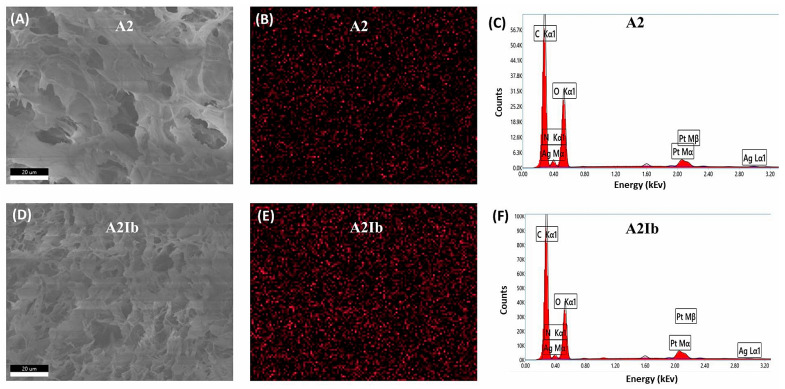
SEM images (**A**,**D**), EDX mapping of Ag (**B**,**E**) and EDX spectra (**C**,**F**) for **A2** and **A2Ib** samples.

The spatial distribution of silver within the hydrogels was investigated by energy-dispersive X-ray (EDX) spectroscopy (Figure 5). The elemental mapping images for silver (Figure 5B,E) showed that AgNPs were uniformly distributed in both samples. This was a result of the homogenous distribution of AgNps coated with CS in the initial polymeric solution subjected to freeze–thawing. Moreover, since the CS-covered AgNPs were involved in physical cross-linking with OA, they remained stable in the matrix after the freeze–thawing, lyophilization, and washing steps. The EDX analysis also confirmed the good stability of the AgNPs even after the drug loading process (sample **A2Ib**, Figure 5F).

The hydrogels in the swollen state were relatively soft and elastic, with compression moduli between 80 and 140 kPa (Table 1). Wound dressings have to mimic the mechanical properties of the skin but also tolerate external stress. Compressive moduli in the range of 100 kPa were proven to be advantageous for keratinocyte adhesion and proliferation [52,53]. Here, the addition of AgNPs and Ib in hydrogels determined a small increase in the elastic modulus, without a significant difference between the values.

**Figure 6:**
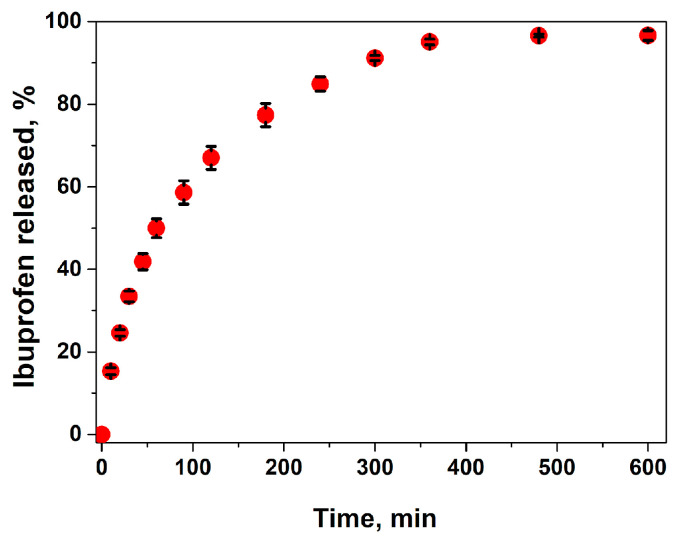
Drug release kinetics from the ibuprofen-loaded composite hydrogel (**A2** sample) in buffer solution pH = 5.5, at 32 °C.

The release profile of ibuprofen in phosphate buffer solution (pH = 5.5) is presented in Figure 6. The composite hydrogel showed a sustained release profile of Ib. In the first 60 min, 48 wt.% of the anti-inflammatory drug was released from the hydrogel, and, after 8 h, almost the entire amount of loaded drug was released. In fact, the Ib release kinetics were influenced by the drug interactions with the polymeric matrix by electrostatic interactions and then by drug diffusion through the polymeric network. The diffusion rate was influenced by steric interactions between the drug and polymeric lattice. Therefore, the higher the swelling degree, the faster the diffusion. In PB at pH = 5.5, the electrostatic interactions between the anionic drug and CS were weakened by the presence of salts from the buffer solution, so the ibuprofen was easily released by diffusion through the porous matrix. The release results are in agreement with the high swelling ratio of the hydrogel, with the swelling equilibrium being attained in the first 2 h (see Figure 4C). The release rate of ibuprofen was higher compared to chitosan–ibuprofen aerogels, where the drug was introduced before the freezing and lyophilization of the chitosan solution [54].

In order to establish the mechanism of ibuprofen release from the hydrogels, the first 60% experimental release data were fitted to zero-order (Equation (1)), Higuchi (Equation (2)) and Korsmeyer–Peppas models (Equation (3)) [55]:

(1) $$M_{t}=k_{0}t$$

(2) $$\frac{M_{t}}{M_{\infty }}=k_{H}t^{\frac{1}{2}}$$

(3) $$\frac{M_{t}}{M_{\infty }}=k_{KP}t^{n}$$

where $M_{t}$ is the amount of drug released at time *t*, $M_{\infty }$ is the total amount of drug contained in the hydrogel, *k* are the kinetic constants (*k*_0_—zero-order constant, *k_H_*—Higuchi constant, *k_KP_*—Korsmeyer–Peppas constant) and *n* is the exponent of release (related to the drug release mechanism).

The results are presented in Table 2. From the correlation coefficients (*R*^2^), it is evident that ibuprofen release does not follow zero-order kinetics, but the experimental data can be fitted to the Higuchi model and even better to the Kosmeyer–Peppas model. The value of the exponential coefficient (*n*) is slightly higher than 0.5 but lower than 1, indicating anomalous transport, meaning that the mechanism of drug release is governed by the diffusion and swelling of the polymeric matrix. In order to approximate the two contribution mechanisms, the Peppas–Sahlin model (Equation (4)) can be applied [55]:

(4) $$\frac{M_{t}}{M_{\infty }}=k_{1}t^{m}+k_{2}t^{2m}$$

where *k*_1_ is a constant that relates to the release rate due to diffusion, *k*_2_ is a constant that corresponds to the release rate due to the relaxation of the polymeric matrix and *m* is a diffusional coefficient in relation to the release system (polymeric matrix and device). The fitting of the experimental data to this equation (Table 2) showed that *k*_2_ had a much lower value than *k*_1_, meaning that the relaxation of the polymeric chains in the hydrogel matrix had an insignificant effect compared to the solvent Fickian diffusion. Considering the final application of the hydrogel for wound dressing, this effect is desirable. The initial pain relief and diminishing of the acute inflammatory response in the early stages of healing will be provided by Ib released in the first hours. Additionally, the hydrogel will maintain controlled moisture at the wound site along with the antibacterial protection assured by the Ag ions.

### 2.2. Evaluation of Antibacterial Activity

**Figure 7:**
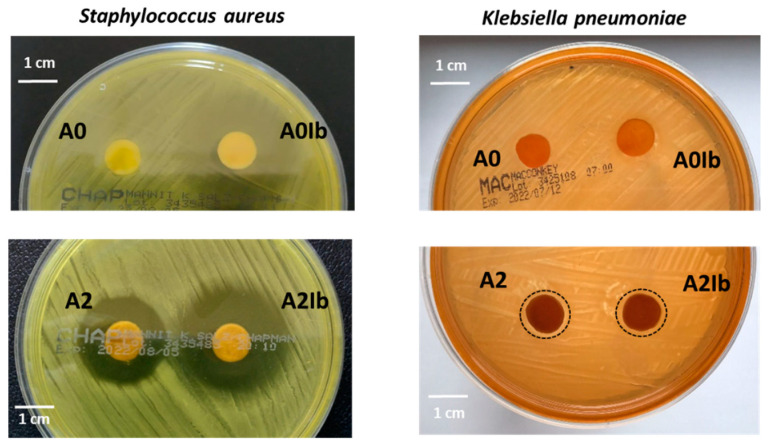
Antimicrobial activity against *S. aureus* and *K. pneumoniae* of ibuprofen-loaded CS/PVA hydrogels without and with AgNPs.

Generally, an increase in the amount of AgNPs amplifies the antimicrobial activity but inherently increases the cytotoxic effect of the materials [14,22,56], so a good balance between these two effects is required. This is the reason for entrapping a relatively small amount of AgNPs in hydrogels (0.3% by weight). The antimicrobial activity of the drug-loaded hydrogels with and without AgNPs was evaluated by the agar disc diffusion method. The diameter of the inhibition zones was used as a measure of the antimicrobial activity. Figure 7 presents the results obtained for the hydrogels tested against two strains: *S. aureus*, known as the predominant species of Gram-positive bacteria found in infected wounds, and *K. pneumoniae*, one of the Gram-negative bacteria isolated from these wounds [57]. No inhibition zones were observed around the **A0** sample. The loading of ibuprofen determined activity against *S. aureus*: the diameter of the inhibition zone for sample **A0Ib** was 32.4 ± 1.2 mm, but this zone was not sufficiently clear (Figure 7). This was not an unexpected result because ibuprofen is known in the literature to have an antimicrobial effect against some microorganisms, including *S. aureus* [58,59].

The entrapment of AgNPs into hydrogels led to the appearance of a clear inhibition zone with a diameter of 24.8 ± 0.4 mm for the **A2** sample placed on the agar plate inoculated with *S. aureus*. The composite hydrogel loaded with ibuprofen—the **A2Ib** sample—showed an improved antibacterial effect (inhibition diameter = 32.6 ± 1.3 mm) due to the presence of both nanoparticles and the drug. The Gram-negative bacterium *K. pneumoniae* was less sensitive to the presence of AgNPs in this low amount, so both the **A2** and **A2Ib** samples determined an inhibition area of only 12 mm in diameter.

### 2.3. In Vivo Stimulant for Wound Healing

**Figure 8:**
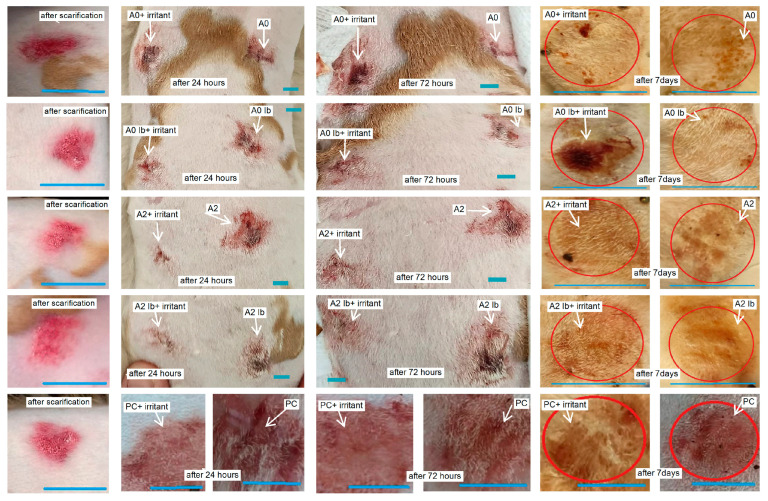
Macroscopic evolution of scarified skin lesions after exposure to different healing hydrogel formulations. Red circles indicate the area of wound healing. White arrows mark the skin lesions and their progressive healing. Blue lines mark the dimensions of lesions and is equal of 1 cm.

At 24 h, the evolution of the lesions was favorable as the healing process had already begun. By 72 h, most of the lesions showed significant improvements, except for group **A2**, which did not receive the irritant. Notably, the lesions treated with **PC+irritant**, **A0+irritant**, **A0Ib+irritant**, **A2+irritant** and **A2Ib+irritant** displayed better progress compared to those without irritants. The most impressive healing results were observed seven days after the rabbits were scarified in groups **A2**, **A2Ib** and **A2Ib+irritant agent**, where the epidermis and hairs had completely recovered, resulting in a fully restored appearance. The subjects in the other groups showed also almost complete recovery, except that small scabs could still be observed (Figure 8). Wound healing is a multifaceted and constantly evolving biological process that involves a coordinated series of cellular and biochemical events, ultimately aiming to restore the structural and functional integrity of damaged tissue [60]. This intricate process comprises four distinct yet interconnected phases, which occur in a temporal and spatial overlap. The initial phase focuses on stopping bleeding and involves the formation of a blood clot to seal the wound. Following hemostasis, the wound site becomes inflamed, involving the activation of immune cells to remove debris and protect against infection. In the proliferation phase, new tissue formation occurs through cell proliferation and migration, which helps to rebuild the damaged tissue. The final phase involves the restructuring and strengthening of the new tissue through the reorganization of collagen fibers and other extracellular matrix components. The hemostasis, inflammation, proliferation and remodeling phases work harmoniously together, ensuring an effective and coordinated process of wound healing [61].

In response to skin injury, pro-inflammatory cytokines play a crucial role by being among the first factors produced and regulating immune cell functions during epithelialization. These cytokines, tumor necrosis factor (TNF) and interleukins IL-6, IL-1 and IL-17, primarily contribute to the inflammatory phase by activating downstream cascades in the wound healing process [62].

**Figure 9:**
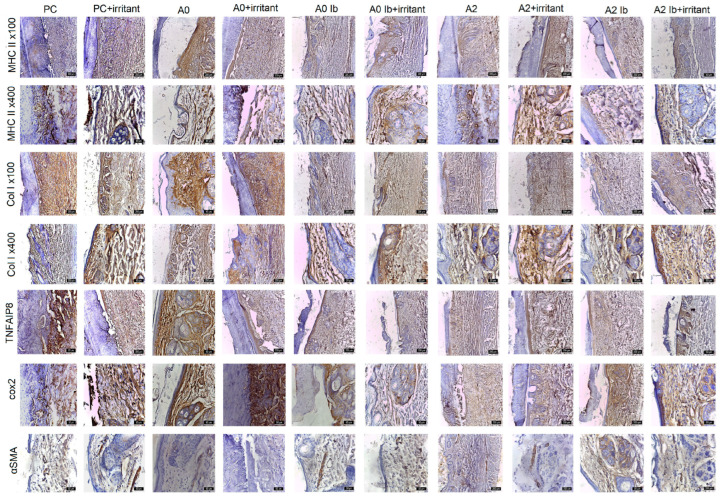
Evolution of markers in scarified skin exposed to different healing gels. IHC staining with anti-CMH II, anti-Col I, anti-TNFIP8, anti-Cox2, anti-SMA; PC—positive control.

The intense positivity of TNF alpha IP8 was observed in groups **PC**, **A0**, **A2+irritant** and **A2Ib+irritant**, with labeling detected in keratinocytes, dermal cells and fibers surrounding developing hair follicles (Figure 9). Furthermore, TNF alpha IP8 is involved in the epithelization phase of wound healing, where it facilitates the mobilization of resident stem/progenitor cells and stimulates cell proliferation and differentiation [63]. TNF-α plays a vital role in regulating wound healing, with its inhibition causing delayed skin regeneration in patients and chronic over-expression negatively impacting skin regeneration [64]. During the inflammatory phase, TNF-α plays a crucial role in synthesizing cell surface adhesion molecules on endothelial cells, facilitating the migration and adhesion of neutrophils to the endothelium. As the wound enters the proliferative phase, TNF-α promotes keratinocyte proliferation and upregulates the expression of intracellular adhesion molecule-1 [65]. Additionally, other cytokines with pro-inflammatory effects also significantly contribute to the wound healing process by recruiting immune cells and stimulating the proliferation and migration of keratinocytes and fibroblasts. IL-1, which is produced by keratinocytes, macrophages and neutrophils, plays a crucial role in preventing wound infection and induces fibroblasts to secrete various factors, including keratinocyte growth factor, fibroblast growth factor-7, granulocyte-macrophage colony-stimulating factor, IL-6 and hepatocyte growth factor [66]. These interactions between fibroblasts and keratinocytes produce a paracrine loop that supports wound healing. Inadequate IL-1 production can lead to the delayed epithelialization of skin lesions [64]. In addition, through autocrine mechanisms, TNF-α stimulates keratinocyte migration and, through paracrine mechanisms, it activates fibroblasts to secrete the FGF family [64]. Moreover, TNF-α-induced apoptosis, whether dependent on or independent of TNFR1, can hinder the production of inflammatory cytokines in keratinocytes, ultimately impeding epidermal differentiation [67]. Lastly, pro-inflammatory cytokines such as TNF, IL-1 and IL-17 play roles in promoting hair follicle regeneration and epithelization during wound healing. IL-17 and IL-1 have the capacity to increase the active γδT cell population, thus boosting the mobilization and proliferation of hair follicle stem cells (hSCs) [68].

During the inflammatory phase of wound healing, the wound is initially sealed by the formation of a fibrin clot, which acts as a temporary matrix. This blood clot provides a skeleton for the migration of immune cells: macrophages, neutrophils, monocytes, mast cells and lymphocytes. These immune cells infiltrate the wound site, removing dead tissue and controlling infection [69]. Keratinocytes in the epidermis, including those in the basal layer and parabasal layer and stem cells associated with developing hair follicles, as well as perivascular stem cells, express MHCII markers. These markers were detected in all experimental groups. It is noteworthy that a higher frequency of MHCII positivity was observed in batches **PC**, **A0** and **A2** (Figure 9), which were associated with the irritant agent.

Following cell proliferation, the wound undergoes remodeling. Fibroblasts are recruited to the wound site, where they play a crucial role in the synthesis and secretion of collagen, a key component in the formation of granulation tissue. The formation of granulation tissue is essential for angiogenesis, which facilitates the transportation of fluids, oxygen, nutrients and immune cells [70]. In the scarified areas covered by hydrogels, a slight increase in collagen deposition was observed. This finding is significant as collagen biosynthesis, deposition and maturation are essential for the wound repair process. Moreover, collagen deposition in wounds contributes to the tensile strength of the resulting scars [71]. Collagen I was detected in all experimental groups, with higher levels of positivity observed in the following, in descending order: **PC**, **A0**, **A0+irritant**, **A0Ib+irritant**, **A2+irritant**, **A2**, **A2Ib+irritant** (Figure 9). In the basal and parabasal layers, collagen fibers and a small number of fibroblasts and keratinocytes were labeled.

COX-2 plays a significant role in various responses to skin injuries, including those caused by injury. Strong positivity of Cox2 was observed in groups **PC+irritant, A0+irritant**, **A0**, **A0Ib** and **A2Ib** (Figure 9). After injury, COX-2 and COX-2 mRNA were mainly expressed in the basal and upper layers of the injured epidermis, which consist of proliferative and migratory cells [72]. Previous studies have also demonstrated the presence of COX-2 immunolabeling after the application of an irritant in the basal layer of the mouse epidermis, whereas normal mouse skin did not show COX-2 immunolabeling [73,74]. The presence of COX-2 and COX-2 mRNA in the endothelial cells of small blood vessels and fibroblast-like cells in granulation tissue were also observed. Moreover, Western blot analysis also confirmed the marked induction of the COX-2 protein at 12 h post-injury, with a continuous rise in concentration from day 1 to day 7. This period is characterized by the significant migration and proliferation of epidermal cells at the wound margins and granulation tissue formation [75]. Especially in the early acute phase, this increased expression of COX-2 may enhance the proliferation and migration of cells involved in re-epithelization and angiogenesis. However, these cells, which proliferate in response to lesions, can also generate migrating epidermal cells. In addition, stem cells are found in the basal layer of the epidermis and are responsible for epidermal maintenance and repair [76]. Therefore, the distinct expression model of the COX-2 protein may be associated with the pattern of epidermal and follicular stem cell distribution. In human non-lesional skin, COX-2 protein expression has been reported in individual epidermal keratinocytes and hair follicles [74].

The process of re-epithelization in wound healing relies on coordinated interactions between inflammatory cells, immune cells, vascular endothelial cells, fibroblasts, keratinocytes and the local microenvironment. The migration and proliferation of keratinocytes are influenced by their interactions with fibroblasts, the extracellular matrix (ECM) and various paracrine factors present at the wound site [77]. During re-epithelization, endogenous epithelial stem cells (EpSCs) that differentiate into epithelial cells play a role in epithelial tissue regeneration. Moreover, keratinocytes at the base of the wound margins migrate under the fibrin clot to actively take part in the re-epithelization process [78].

Epithelialization, which is crucial for wound healing, is achieved through the activation, migration and proliferation of epidermal stem cells, leading to the restoration of a functional layer of keratinocytes [79]. Additionally, the remodeling phase involves the restructuring of the extracellular matrix, which can result in scar formation [80]. Stem cells have a unique ability to renew and differentiate into different cell types [81]. In the context of cutaneous wound healing, these stem cells play a vital role by repairing damaged tissue, enhancing the migration of fibroblasts and keratinocytes, promoting angiogenesis and facilitating the production of collagen and elastin [82]. Keratinocytes are the primary cellular components of the epidermis and originate from epithelial stem cells. EpSCs are located mainly in the basal layer of the interfollicular epidermis and the bulb of the hair follicle [83,84]. Following the formation of an epidermal lesion, epithelial stem cells in both deposits give rise to migrating keratinocytes that contribute to the re-epithelization of the wound [85,86]. Studies have shown that EpSCs in the interfollicular epidermis are significant contributors to long-term epidermal repair [87]. Although stem cells from the hair bulb initially migrate into the interfollicular epidermis to regenerate the epidermis following injury, this effect is temporary, indicating that hair follicles and EpSCs primarily facilitate the early stages of healing in superficial epidermal wounds, while their contribution may be less prominent in larger or more challenging-to-heal wounds where spontaneous re-epithelization is compromised [88,89]. Overall, the process of re-epithelization involves the activation, proliferation, migration and differentiation of keratinocytes to form a new epithelium and restore the integrity of the underlying dermal structures [90].

Upon complete coverage of the wound area, contact inhibition mechanisms halt the migration of keratinocytes and initiate their differentiation into keratinizing squamous epidermal stratified cells [91]. In the experimental groups observed at 7 days after scarification, granulation tissue was observed in the **A0+irritant** and **A2** batches (Figure 9). The granulation tissue was only covered by thin epithelial lamellae in the peripheral transition zones. In the other batches, partial regeneration of the epithelium was observed in the lesion area, characterized by flattened cells arranged in one to three rows, but without the presence of hair follicles. The surrounding skin displayed a normal appearance, with mature hair follicles interspersed with developing ones in the dermis. Type I collagen fibers in the papillary layer of the dermis were oriented parallel to the skin surface. The use of the irritant agent had a stimulating effect, as indicated by the more intense expression of all the markers used in these groups.

During the re-epithelization process, keratinocytes play a crucial role in restoring the epidermis by increasing their migration and undergoing mitosis at the edges of the wound, ultimately integrating into the epidermal layer. Furthermore, myofibroblasts migrate beneath the wound site to facilitate wound closure [92]. The evaluation of the repair process involved the examination of myofibrillar populations using an alpha SMA antibody. Smooth muscle fibers of the arrector pili muscle stained positive for SMA in all groups (Figure 9). In certain groups, such as **A2Ib**, **A2Ib+irritant**, **A0Ib**, **A0Ib+irritant**, **PC** and **PC+irritant**, a small number of myofibroblasts were observed. The intense expression of myofibroblasts suggests that the injured margins were in close proximity, indicating ongoing re-epithelization.

In summary, the healing process involves a complex interplay of cellular and molecular events, involving the temporary formation of a wound bed matrix, the proliferation and migration of keratinocytes, the reconstitution of the DEJ, collagen deposition and the formation of a functional epidermis. Various factors, including growth factors, cytokines and matrix metalloproteinases, contribute to the successful completion of re-epithelization. Keratinocyte activation, migration and differentiation are orchestrated by a range of signals and interactions with neighboring cells. Fibroblasts play a role in wound closure, and collagen deposition provides strength to the resulting scars. Prostaglandin biosynthesis mediated by COX-2 is crucial for skin wound healing. Overall, the intricate cellular and molecular processes involved in epithelialization are essential for the successful closure of wounds.

## 3. Conclusions

The healing of skin wounds in mammals is an intricate journey comprising several interconnected phases. These include the formation of blood clots, the initiation of an inflammatory response, the re-establishment of a new layer of epithelium on the wound surface (known as re-epithelization), granulation tissue formation, neovascularization and further remodeling of the tissue. These stages rely on a complex interplay of cellular and molecular processes, where various factors such as growth factors, cytokines, matrix metalloproteinases, cellular receptors and components of the extracellular matrix act as important regulators. The orchestrated coordination of these processes is essential for the effective closure of wounds.

The results clearly demonstrate the potential of composite hydrogels with embedded silver nanoparticles and ibuprofen to be used for wound dressing. First, the hydrogel showed relevant antibacterial activity against relevant pathogens (*S. aureus* and *K. pneumoniae*); second, it acted as a stimulus in the healing process, supporting the healing process of inflammation, the proliferative process and finally the reshaping of the damaged tissue. Following macroscopic and microscopic examination, the **A2** and **A2Ib** products yielded the best healing results.

## 4. Materials and Methods

### 4.1. Materials

Chitosan (CS, Mv = 240,000 g/mol, deacetylation degree = 80%), poly(vinyl alcohol) (PVA, Mowiol^®^ 20–98, 98–98.8 mol% hydrolysis, Mw ~ 125,000 g/mol) and ibuprofen sodium salt (Ib, 98% purity) were purchased from Sigma Aldrich Co. (St. Louis, MO, USA).

CS-capped AgNPs (CS-AgNPs), containing 18 wt% Ag and with a size between 4 and 22 nm, were obtained according to our previous method [14].

Oxalic acid (OA, anhydrous 98%) was from Alfa Aesar—ThermoFisher (Kandel, Germany). Distilled water (4 µS/cm conductivity) was used for all the experiments.

### 4.2. Preparation of Hydrogels with or without AgNPs

Exact amounts of CS, CS-AgNPs, oxalic acid and water were mixed in order to obtain a solution containing 3.84 wt.% CS, 0.126 wt.% CS-AgNPs and 3.94 wt.% oxalic acid. The mixture was stirred for 20 h at room temperature and then heated at 60 °C for 30 min in order to ensure the breaking of hydrogen bonds and complete dissolution of CS. Separately, a 10 wt.% PVA solution was obtained by solving PVA in water at 80 °C for 2 h. After cooling, the CS and PVA solutions were mixed in a 2.6:1 gravimetric ratio. The obtained mixture, containing 5.6 wt.% polymer and a gravimetric ratio of 1:1 between CS and PVA, was stirred for 4 h, degassed and then poured onto Petri dishes (5 cm diameter) in order to obtain hydrogels in form of films with 2 mm height. The samples were subjected to 7 freeze–thawing cycles, with one cycle consisting of 18 h at −20 °C and 6 h at room temperature. After the last freezing step, the samples were dried by lyophilization for 48 h at −57 °C and 0.05 mbar, with an Alpha 1-2 LD Martin Christ freeze-dryer (Osterode am Harz, Germany).

The hydrogel samples without AgNPs were prepared using the same procedure, except that the content of CS and oxalic acid in the first solution was 3.94 wt.% for each one. All hydrogel samples were extensively washed with distilled water for 3 days, replacing the water every day, in order to remove the leachable polymeric chains and oxalic acid that were not involved in the physical cross-linking; then, they were dried again by freeze-drying. The hydrogels without AgNPs were named **A0**, and the samples with embedded metallic nanoparticles were coded **A2**.

### 4.3. Preparation of Ibuprofen-Loaded Hydrogels

The ibuprofen-loaded hydrogels were prepared by the immersion of the dried hydrogel into a 4 mg/mL Ib solution (0.5:1 gravimetric ratio between Ib and hydrogel) for 48 h. At the end of the experiment, the hydrogel was taken out from the drug solution, washed with water in order to remove the excess ibuprofen and then dried by freeze-drying. The drug loading amount was calculated from the concentrations of the drug solution before and after absorption into the hydrogel. The ibuprofen concentration was determined by UV spectroscopy using an Evolution 201 UV–Visible Spectrometer (Thermo Fisher Scientific, Waltham, MA, USA). The calibration curve of Ib was previously constructed at λ = 264 nm. The drug-loaded hydrogels were coded **A0Ib** (hydrogels without AgNPs) and **A2Ib** (hydrogels with AgNPs).

### 4.4. Physico-Chemical Characterization of the Hydrogels

The Fourier transform infrared spectroscopy (FT-IR) analysis of samples was performed on a VERTEX 70 FT-IR spectrometer (Bruker, Ettlingen, Germany) in the range of 4000 and 400 cm^−1^ at a resolution of 4 cm^−1^. The freeze-dried hydrogels were ground and mixed with KBr for the obtaining of the tablets used in FT-IR spectroscopy.

The morphology of the hydrogels was evaluated by scanning electron microscopy (SEM) with a Quanta 200 electron microscope (FEI Company, Brno, Czech Republic).

The energy-dispersive X-ray analysis (EDX) was performed using a Verious G4 UC scanning electron microscope (Thermo Scientific, Brno-Černovice, Czech Republic) equipped with an EDX analyzer, the Octane Elect Super SDD detector. A cross-section of the composite hydrogel was coated with 6 nm platinum before the examination, and the investigations were performed in high vacuum mode at an accelerating voltage of 10 kV.

The content of CS in hydrogels was determined by the ninhydrin assay [93,94]. Briefly, 5 mL ninhydrin reactive (0.8 g ninhydrin, 0.12 g hydrindantin, 10 mL of 4M lithium acetate buffer with pH = 5.2, and 30 mL DMSO) was added to 5 mg hydrogel swollen in 5 mL acetic acid 0.5%. The mixture was heated in boiling water for 30 min. The obtained blue solution was cooled down and diluted with ethanol:water (1:1), and the UV–Vis spectra were recorded. The CS concentration was determined from the absorbance at 570 nm, using a previously obtained calibration curve.

The silver content in the hydrogels was determined by atomic absorption spectroscopy. The hydrogel was first digested in 65% HNO_3_ for 24 h. The obtained solution was diluted with water and the exact silver concentration was measured with a ContrAA 800 spectrometer (Analytik Jena, Jena, Germany) in an air/acetylene flame at 328 nm.

### 4.5. Swelling Ratio

The swelling behavior of the hydrogels was analyzed in simulated skin condition media, phosphate buffer (PB), pH = 5.5. The dried hydrogels were weighed ($w_{d}$), introduced into the buffer solution and, after different time intervals, withdrawn, blotted with filter paper and weighed again ($w_{t}$). The swelling ratio (*SR*) was calculated according to Equation (5):

(5) $$SR=\frac{w_{t}-w_{d}}{w_{d}}$$

### 4.6. Mechanical Properties

The mechanical properties of the hydrogels in the swollen state were studied by compression tests using a Brookfield Texture PRO CT3 texture analyzer (Brookfield Engineering Laboratories Inc., Middleborough, MA, USA). The hydrogel samples were prepared especially for these measurements (around 16 mm diameter, 6 mm high) and the exact dimensions were measured with a digital caliper. The compression tests were performed with a speed of 0.2 mm/s. The elastic modulus was calculated from the slope of the straight-line portion of the stress–strain curve (between 3 and 20% deformation).

### 4.7. In Vitro Drug Release Studies

The drug release performance of the ibuprofen-loaded hydrogels was examined in phosphate buffer pH = 5.5 at 32 °C (simulated skin conditions) using a static experimental model [22]. The drug-loaded hydrogel samples (20 mg) were immersed in 3 mL PB solution and, at different time intervals, 2 mL of the release medium was removed and replaced with the same volume of fresh buffer. The concentration of ibuprofen in the collected solutions was measured by UV–Vis spectrophotometry.

### 4.8. In Vitro Antimicrobial Assay

The antimicrobial activity of the hydrogels with and without AgNPs loaded or not with Ib was tested by the Kirby–Bauer disc diffusion susceptibility method [95]. The effect of the hydrogels against two pathogens was tested: *Staphylococcus aureus* ATCC 25923 and *Klebsiella pneumoniae* ATCC BAA-1705. The hydrogel discs (10 mm diameter) were hydrated and then placed on agar plates previously seeded with each strain. After incubation for 24 h at 37 °C, the diameter of the inhibition zone was measured. Three parallel samples were adopted for each experiment.

### 4.9. Animal Experiment

In vivo testing was performed at Iasi University of Life Sciences “Ion Ionescu de la Brad”, Faculty of Veterinary Medicine Iasi, in the Histology and Embryology Laboratory and at the biobasis. The aim of the study was to investigate both the compatibility of the new drug products (**A0**, **A0Ib**, **A2**, **A2Ib**) and the therapeutic healing effect in rabbit skin.

The biological material consisted of 16 rabbits, of common breed, with an average age of 1 year, divided into 2 large groups: a group without treatment applied to a single rabbit (**PC** and **PC+irritant**) and a second large group consisting of 5 smaller groups of 3 animals each, a control group (**A0**) and four experimental groups (**A0+irritant, A0Ib, A2+irritant, A2Ib**). In the rabbit without treatment, the left side was scarified and then covered with a sterile dressing, and the right side was scarified and treated with irritant solution only on the first day and covered with a sterile dressing as well. Animals were housed and handled in accordance with the European Animal Welfare Act under the supervision of veterinarians and monitored for any clinical, attitudinal or behavioral changes that would indicate disease. The ethical clearance of the present study was granted under no. 181/01.03.2021.

The animals in the experiment were analogous in terms of age, breed, body weight and state of health. They were housed under similar conditions of temperature, humidity, lighting and care. Animals were then placed under sedation by the intramuscular injection of xylazine and ketamine. Two areas of 1.5/1.5 cm each on the dorsal thoracic side were obtained by scarification for each animal. On one of the areas, the healing effect of the complex was monitored. The other area was treated with 0.5 mL of 95% ethyl alcohol for irritation and acceleration of the healing effect. The used dressings were changed every 1 day (24 h) and 3 days (72 h). During the whole experiment, rabbits were closely monitored and evaluated. Seven days after scarification, rabbits were sedated with xylazine and ketamine by intramuscular injection and then sacrificed. Skin samples were then taken and fixed with 10% formalin solution for 24 h. After one day, samples were dehydrated with ethyl alcohol, clarified with xylene and embedded in paraffin. Samples were sectioned at 4 µm and subjected to HE and IHC staining with the following markers: tumor necrosis factor-α-induced protein-8 (TNFIP8), COX-2, MHC II, collagen I (Col I) and α-SMA.

## Figures and Tables

**Table 1:** The chemical composition of the hydrogels and their compression modulus.

Sample Code	CS Content, wt.%		Ag Content, mg/g		Ibuprofen Loading, mg/g	Compression Modulus, kPa
	Theoretical	Ninhydrin Assay	Theoretical	AAS		
**A0**	33	45.2 ± 1.6	-	-		91 ± 2
**A0Ib**	26	n.d.	-	-	270.5 ± 3.5	n.d.
**A2**	33	n.d.	3.0	2.98 ± 0.03	-	117 ± 3
**A2Ib**	26.1	36.4 ± 0.4	2.37	2.25 ± 0.06	264 ± 2	132 ± 6

n.d.—not determined.

**Table 2:** Parameters and correlation coefficients (*R*^2^) for different release models.

Zero Order		Higuchi		Kosmeyer–Peppas		Peppas–Sahlin	
*k*_0_ (min^−1^)	1.93	*k_H_* (min^−0.5^)	6.13	*k_KP_* (min^−0.57^)	4.60	*k*_1_ (min^−0.45^)	5.32
				*n*	0.57	*k*_2_ (min^−0.9^)	0.35
*R* ^2^	0.903	*R^2^*	0.987	*R* ^2^	0.993	*R* ^2^	0.980

## Data Availability

The data presented in this study are available on request from the corresponding author.

## References

[1] Falanga V. (2005). Wound Healing and Its Impairment in the Diabetic Foot. Lancet.

[2] Shukla S.K., Sharma A.K., Gupta V., Yashavarddhan M.H. (2019). Pharmacological Control of Inflammation in Wound Healing. J. Tissue Viability.

[3] Schultz G.S., Sibbald R.G., Falanga V., Ayello E.A., Dowsett C., Harding K., Romanelli M., Stacey M.C., Teot L., Vanscheidt W. (2003). Wound Bed Preparation: A Systematic Approach to Wound Management. Wound Repair Regen..

[4] Tavakoli S., Klar A.S. (2020). Advanced Hydrogels as Wound Dressings. Biomolecules.

[5] Koehler J., Brandl F.P., Goepferich A.M. (2018). Hydrogel Wound Dressings for Bioactive Treatment of Acute and Chronic Wounds. Eur. Polym. J..

[6] Popescu I., Turtoi M., Suflet D.M., Dinu M.V., Darie-Nita R.N., Anghelache M., Calin M., Constantin M. (2021). Alginate/Poloxamer Hydrogel Obtained by Thiol-Acrylate Photopolymerization for the Alleviation of the Inflammatory Response of Human Keratinocytes. Int. J. Biol. Macromol..

[7] Mohan K., Ganesan A.R., Muralisankar T., Jayakumar R., Sathishkumar P., Uthayakumar V., Chandirasekar R., Revathi N. (2020). Recent Insights into the Extraction, Characterization, and Bioactivities of Chitin and Chitosan from Insects. Trends Food Sci. Technol..

[8] Feng P., Luo Y., Ke C., Qiu H., Wang W., Zhu Y., Hou R., Xu L., Wu S. (2021). Chitosan-Based Functional Materials for Skin Wound Repair: Mechanisms and Applications. Front. Bioeng. Biotechnol..

[9] Jin S.G. (2022). Production and Application of Biomaterials Based on Polyvinyl Alcohol (PVA) as Wound Dressing. Chem. Asian J..

[10] Adelnia H., Ensandoost R., Shebbrin Moonshi S., Gavgani J.N., Vasafi E.I., Ta H.T. (2022). Freeze/Thawed Polyvinyl Alcohol Hydrogels: Present, Past and Future. Eur. Polym. J..

[11] Cascone M.G., Maltinti S., Barbani N., Laus M. (1999). Effect of chitosan and dextran on the properties of poly(vinyl alcohol) hydrogels. J. Mater. Sci. Mater. Med..

[12] Figueroa-Pizano M.D., Vélaz I., Peñas F.J., Zavala-Rivera P., Rosas-Durazo A.J., Maldonado-Arce A.D., Martínez-Barbosa M.E. (2018). Effect of Freeze-Thawing Conditions for Preparation of Chitosan-Poly (Vinyl Alcohol) Hydrogels and Drug Release Studies. Carbohydr. Polym..

[13] Mathews D.T., Birney Y.A., Cahill P.A., McGuinness G.B. (2008). Mechanical and Morphological Characteristics of Poly(Vinyl Alcohol)/Chitosan Hydrogels. J. Appl. Polym. Sci..

[14] Popescu I., Constantin M., Pelin I.M., Suflet D.M., Ichim D.L., Daraba O.M., Fundueanu G. (2022). Eco-Friendly Synthesized PVA/Chitosan/Oxalic Acid Nanocomposite Hydrogels Embedding Silver Nanoparticles as Antibacterial Materials. Gels.

[15] Mendes C., Thirupathi A., Corrêa M.E.A.B., Gu Y., Silveira P.C.L. (2022). The Use of Metallic Nanoparticles in Wound Healing: New Perspectives. Int. J. Mol. Sci..

[16] Yougbaré S., Mutalik C., Okoro G., Lin I.-H., Krisnawati D.I., Jazidie A., Nuh M., Chang C.-C., Kuo T.-R. (2021). Emerging Trends in Nanomaterials for Antibacterial Applications. Int. J. Nanomed..

[17] Konop M., Damps T., Misicka A., Rudnicka L. (2016). Certain Aspects of Silver and Silver Nanoparticles in Wound Care: A Minireview. J. Nanomater..

[18] Zakia M., Koo J.M., Kim D., Ji K., Huh P., Yoon J., Yoo S.I. (2020). Development of Silver Nanoparticle-Based Hydrogel Composites for Antimicrobial Activity. Green Chem. Lett. Rev..

[19] Hiep N.T., Khon H.C., Niem V.V.T., Toi V.V., Ngoc Quyen T., Hai N.D., Ngoc Tuan Anh M. (2016). Microwave-Assisted Synthesis of Chitosan/Polyvinyl Alcohol Silver Nanoparticles Gel for Wound Dressing Applications. Int. J. Polym. Sci..

[20] Suflet D.M., Popescu I., Pelin I.M., Ichim D.L., Daraba O.M., Constantin M., Fundueanu G. (2021). Dual Cross-Linked Chitosan/PVA Hydrogels Containing Silver Nanoparticles with Antimicrobial Properties. Pharmaceutics.

[21] Cadinoiu A.N., Rata D.M., Daraba O.M., Ichim D.L., Popescu I., Solcan C., Solcan G. (2022). Silver Nanoparticles Biocomposite Films with Antimicrobial Activity: In Vitro and In Vivo Tests. Int. J. Mol. Sci..

[22] Constantin M., Lupei M., Bucatariu S.-M., Pelin I.M., Doroftei F., Ichim D.L., Daraba O.M., Fundueanu G. (2022). PVA/Chitosan Thin Films Containing Silver Nanoparticles and Ibuprofen for the Treatment of Periodontal Disease. Polymers.

[23] Orlando B.J., Lucido M.J., Malkowski M.G. (2015). The Structure of Ibuprofen Bound to Cyclooxygenase-2. J. Struct. Biol..

[24] Lisboa F.A., Bradley M.J., Hueman M.T., Schobel S.A., Gaucher B.J., Styrmisdottir E.L., Potter B.K., Forsberg J.A., Elster E.A. (2017). Nonsteroidal Anti-Inflammatory Drugs May Affect Cytokine Response and Benefit Healing of Combat-Related Extremity Wounds. Surgery.

[25] Morgado P.I., Miguel S.P., Correia I.J., Aguiar-Ricardo A. (2017). Ibuprofen Loaded PVA/Chitosan Membranes: A Highly Efficient Strategy towards an Improved Skin Wound Healing. Carbohydr. Polym..

[26] Akgun A.E., Alkin M. (2023). Pain Management with Topical Ibuprofen in Partial-Thickness Burn Wounds and Effects on Wound Healing: A Prospective Randomized Clinical Study. Wound Manag. Prev..

[27] Wang X.-H., Su T., Zhao J., Wu Z., Wang D., Zhang W.-N., Wu Q.-X., Chen Y. (2020). Fabrication of Polysaccharides-Based Hydrogel Films for Transdermal Sustained Delivery of Ibuprofen. Cellulose.

[28] Uchiyama M.K., Hebeda C.B., Sandri S., de Paula-Silva M., Romano M., Cardoso R.M., Toma S.H., Araki K., Farsky S.H. (2021). In Vivo Evaluation of Toxicity and Anti-Inflammatory Activity of Iron Oxide Nanoparticles Conjugated with Ibuprofen. Nanomedicine.

[29] Theochari I., Mitsou E., Nikolic I., Ilic T., Dobricic V., Pletsa V., Savic S., Xenakis A., Papadimitriou V. (2021). Colloidal Nanodispersions for the Topical Delivery of Ibuprofen: Structure, Dynamics and Bioperformances. J. Mol. Liq..

[30] Choudhary N., Singh A.P. (2021). Transdermal Drug Delivery System: A Review. Indian J. Pharm. Pharmacol..

[31] Anantrao J.H., Nath P.A., Nivrutti P.R. (2021). Drug Penetration Enhancement Techniques in Transdermal Drug Delivery System: A Review. J. Pharm. Res. Int..

[32] Chu T., Wang C., Wang J., Wang H., Geng D., Wu C., Zhao L., Zhao L. (2020). Chiral 4-*O*-Acylterpineol as Transdermal Permeation Enhancers: Insights of the Enhancement Mechanisms of a Transdermal Enantioselective Delivery System for Flurbiprofen. Drug Deliv..

[33] Fukuta T., Oshima Y., Michiue K., Tanaka D., Kogure K. (2020). Non-Invasive Delivery of Biological Macromolecular Drugs into the Skin by Iontophoresis and Its Application to Psoriasis Treatment. J. Control. Release.

[34] Hu Z., Meduri C.S., Ingrole R.S.J., Gill H.S., Kumar G. (2020). Solid and Hollow Metallic Glass Microneedles for Transdermal Drug-Delivery. Appl. Phys. Lett..

[35] Sabri A., Ogilvie J., McKenna J., Segal J., Scurr D., Marlow M. (2020). Intradermal Delivery of an Immunomodulator for Basal Cell Carcinoma; Expanding the Mechanistic Insight into Solid Microneedle-Enhanced Delivery of Hydrophobic Molecules. Mol. Pharm..

[36] Yang J., Li Y., Ye R., Zheng Y., Li X., Chen Y., Xie X., Jiang L. (2020). Smartphone-Powered Iontophoresis-Microneedle Array Patch for Controlled Transdermal Delivery. Microsyst. Nanoeng..

[37] Kashyap A., Das A., Ahmed A.B. (2020). Formulation and Evaluation of Transdermal Topical Gel of Ibuprofen. J. Drug Deliv. Ther..

[38] Xia M., Tian C., Liu L., Hu R., Gui S., Chu X. (2020). Transdermal Administration of Ibuprofen-Loaded Gel: Preparation, Pharmacokinetic Profile, and Tissue Distribution. AAPS PharmSciTech.

[39] Yadav E., Khatana A.K., Sebastian S., Gupta M.K. (2021). DAP Derived Fatty Acid Amide Organogelators as Novel Carrier for Drug Incorporation and PH-Responsive Release. New J. Chem..

[40] Ramöller I., McAlister E., Bogan A., Cordeiro A., Donnelly R. (2020). Novel Design Approaches in the Fabrication of Polymeric Microarray Patches via Micromoulding. Micromachines.

[41] Jaber N., Al-Akayleh F., Abdel-Rahem R.A., Al-Remawi M. (2021). Characterization Ex Vivo Skin Permeation and Pharmacological Studies of Ibuprofen Lysinate-Chitosan-Gold Nanoparticles. J. Drug Deliv. Sci. Technol..

[42] Lee Y.-S., Wysocki A., Warburton D., Tuan T.-L. (2012). Wound Healing in Development. Birth Defects Res. Part C Embryo Today Rev..

[43] Ashcroft G.S., Yang X., Glick A.B., Weinstein M., Letterio J.J., Mizel D.E., Anzano M., Greenwell-Wild T., Wahl S.M., Deng C. (1999). Mice Lacking Smad3 Show Accelerated Wound Healing and an Impaired Local Inflammatory Response. Nat. Cell Biol..

[44] Faa G., Gerosa C., Fanni D., Monga G., Zaffanello M., Van Eyken P., Fanos V. (2012). Morphogenesis and Molecular Mechanisms Involved in Human Kidney Development. J. Cell. Physiol..

[45] Hsu M., Peled Z.M., Chin G.S., Liu W., Longaker M.T. (2001). Ontogeny of Expression of Transforming Growth Factor-Β1 (TGF-Β1), TGF-Β3, and TGF-β Receptors I and II in Fetal Rat Fibroblasts and Skin. Plast. Reconstr. Surg..

[46] Gros J., Hu J.K.-H., Vinegoni C., Feruglio P.F., Weissleder R., Tabin C.J. (2010). WNT5A/JNK and FGF/MAPK Pathways Regulate the Cellular Events Shaping the Vertebrate Limb Bud. Curr. Biol..

[47] Ihara S., Motobayashi Y., Nagao E., Kistler A. (1990). Ontogenetic Transition of Wound Healing Pattern in Rat Skin Occurring at the Fetal Stage. Development.

[48] Sailakshmi G., Mitra T., Chatterjee S., Gnanamani A. (2013). Engineering Chitosan Using α, ω-Dicarboxylic Acids—An Approach to Improve the Mechanical Strength and Thermal Stability. J. Biomater. Nanobiotechnol..

[49] Ghosh A., Ali M.A. (2012). Studies on Physicochemical Characteristics of Chitosan Derivatives with Dicarboxylic Acids. J. Mater. Sci..

[50] Acharya M., Mishra S., Sahoo R.N., Mallick S. (2017). Infrared Spectroscopy for Analysis of Co-Processed Ibuprofen and Magnesium Trisilicate at Milling and Freeze Drying. Acta Chim. Slov..

[51] Holloway J.L., Lowman A.M., Palmese G.R. (2013). The Role of Crystallization and Phase Separation in the Formation of Physically Cross-Linked PVA Hydrogels. Soft Matter.

[52] Trappmann B., Gautrot J.E., Connelly J.T., Strange D.G.T., Li Y., Oyen M.L., Cohen Stuart M.A., Boehm H., Li B., Vogel V. (2012). Extracellular-Matrix Tethering Regulates Stem-Cell Fate. Nat. Mater..

[53] Zhao X., Lang Q., Yildirimer L., Lin Z.Y., Cui W., Annabi N., Ng K.W., Dokmeci M.R., Ghaemmaghami A.M., Khademhosseini A. (2016). Photocrosslinkable Gelatin Hydrogel for Epidermal Tissue Engineering. Adv. Healthc. Mater..

[54] Li C., Wang K., Xie D. (2021). Green Fabrication and Release Mechanisms of PH-Sensitive Chitosan–Ibuprofen Aerogels for Controlled Transdermal Delivery of Ibuprofen. Front. Chem..

[55] Bruschi M.L. (2015). Strategies to Modify the Drug Release from Pharmaceutical Systems.

[56] Yu Z., Wang W., Dhital R., Kong F., Lin M., Mustapha A. (2019). Antimicrobial Effect and Toxicity of Cellulose Nanofibril/Silver Nanoparticle Nanocomposites Prepared by an Ultraviolet Irradiation Method. Colloids Surf. B Biointerfaces.

[57] Puca V., Marulli R.Z., Grande R., Vitale I., Niro A., Molinaro G., Prezioso S., Muraro R., Di Giovanni P. (2021). Microbial Species Isolated from Infected Wounds and Antimicrobial Resistance Analysis: Data Emerging from a Three-Years Retrospective Study. Antibiotics.

[58] Elvers K.T., Wright S.J.L. (1995). Antibacterial Activity of the Anti-Inflammatory Compound Ibuprofen. Lett. Appl. Microbiol..

[59] AL-Janabi A.A.H. (2010). In Vitro Antibacterial Activity of Ibuprofen and Acetaminophen. J. Glob. Infect Dis..

[60] Salas-Oropeza J., Jimenez-Estrada M., Perez-Torres A., Castell-Rodriguez A.E., Becerril-Millan R., Rodriguez-Monroy M.A., Jarquin-Yañez K., Canales-Martinez M.M. (2021). Wound Healing Activity of α-Pinene and α-Phellandrene. Molecules.

[61] Boateng J.S., Matthews K.H., Stevens H.N.E., Eccleston G.M. (2008). Wound Healing Dressings and Drug Delivery Systems: A Review. J. Pharm. Sci..

[62] Kanji S., Das H. (2017). Advances of Stem Cell Therapeutics in Cutaneous Wound Healing and Regeneration. Mediat. Inflamm..

[63] Larouche J., Sheoran S., Maruyama K., Martino M.M. (2018). Immune Regulation of Skin Wound Healing: Mechanisms and Novel Therapeutic Targets. Adv. Wound Care.

[64] Nosenko M.A., Ambaryan S.G., Drutskaya M.S. (2019). Proinflammatory cytokines and skin wound healing in mice. Mol. Biol..

[65] Shinozaki M., Okada Y., Kitano A., Ikeda K., Saika S., Shinozaki M. (2009). Impaired Cutaneous Wound Healing with Excess Granulation Tissue Formation in TNFα-Null Mice. Arch. Dermatol. Res..

[66] Hu Y., Liang D., Li X., Liu H.-H., Zhang X., Zheng M., Dill D., Shi X., Qiao Y., Yeomans D. (2010). The Role of Interleukin-1 in Wound Biology. Part II: In Vivo and Human Translational Studies. Anesth. Analg..

[67] Piao X., Miura R., Miyake S., Komazawa-Sakon S., Koike M., Shindo R., Takeda J., Hasegawa A., Abe R., Nishiyama C. (2019). Blockade of TNF Receptor Superfamily 1 (TNFR1)–Dependent and TNFR1-Independent Cell Death Is Crucial for Normal Epidermal Differentiation. J. Allergy Clin. Immunol..

[68] Lee P., Gund R., Dutta A., Pincha N., Rana I., Ghosh S., Witherden D., Kandyba E., MacLeod A., Kobielak K. (2017). Stimulation of Hair Follicle Stem Cell Proliferation through an IL-1 Dependent Activation of ΓδT-Cells. eLife.

[69] Martin P., Nunan R. (2015). Cellular and Molecular Mechanisms of Repair in Acute and Chronic Wound Healing. Br. J. Dermatol..

[70] Sorg H., Tilkorn D.J., Hager S., Hauser J., Mirastschijski U. (2017). Skin Wound Healing: An Update on the Current Knowledge and Concepts. Eur. Surg. Res..

[71] Karakaya S., Süntar I., Yakinci O.F., Sytar O., Ceribasi S., Dursunoglu B., Ozbek H., Guvenalp Z. (2020). In Vivo Bioactivity Assessment on Epilobium Species: A Particular Focus on Epilobium Angustifolium and Its Components on Enzymes Connected with the Healing Process. J. Ethnopharmacol..

[72] Garlick J.A., Taichman L.B. (1994). Fate of Human Keratinocytes during Reepithelialization in an Organotypic Culture Model. Lab. Investig..

[73] Leong J., Hughes-Fulford M., Rakhlin N., Habib A., Maclouf J., Goldyne M.E. (1996). Cyclooxygenases in Human and Mouse Skin and Cultured Human Keratinocytes: Association of COX-2 Expression with Human Keratinocyte Differentiation. Exp. Cell Res..

[74] Müller-Decker K., Kopp-Schneider A., Marks F., Seibert K., Fürstenberger G. (1998). Localization of Prostaglandin H Synthase Isoenzymes in Murine Epidermal Tumors: Suppression of Skin Tumor Promotion by Inhibition of Prostaglandin H Synthase-2. Mol. Carcinog..

[75] Clark R.A.F. (1993). Basics of Cutaneous Wound Repair. J. Dermatol. Surg. Oncol..

[76] Jones P.H., Harper S., Watt F.M. (1995). Stem Cell Patterning and Fate in Human Epidermis. Cell.

[77] El Ghalbzouri A., Hensbergen P., Gibbs S., Kempenaar J., van der Schors R., Ponec M. (2004). Fibroblasts Facilitate Re-Epithelialization in Wounded Human Skin Equivalents. Lab. Investig..

[78] Hameedaldeen A., Liu J., Batres A., Graves G., Graves D. (2014). FOXO1, TGF-β Regulation and Wound Healing. Int. J. Mol. Sci..

[79] Rousselle P., Braye F., Dayan G. (2019). Re-Epithelialization of Adult Skin Wounds: Cellular Mechanisms and Therapeutic Strategies. Adv. Drug Deliv. Rev..

[80] Profyris C., Tziotzios C., Do Vale I. (2012). Cutaneous Scarring: Pathophysiology, Molecular Mechanisms, and Scar Reduction Therapeutics. J. Am. Acad. Dermatol..

[81] Yang R., Liu F., Wang J., Chen X., Xie J., Xiong K. (2019). Epidermal Stem Cells in Wound Healing and Their Clinical Applications. Stem Cell Res. Ther..

[82] Kucharzewski M., Rojczyk E., Wilemska-Kucharzewska K., Wilk R., Hudecki J., Los M.J. (2019). Novel Trends in Application of Stem Cells in Skin Wound Healing. Eur. J. Pharmacol..

[83] Pastar I., Stojadinovic O., Yin N.C., Ramirez H., Nusbaum A.G., Sawaya A., Patel S.B., Khalid L., Isseroff R.R., Tomic-Canic M. (2014). Epithelialization in Wound Healing: A Comprehensive Review. Adv. Wound Care.

[84] Lau K., Paus R., Tiede S., Day P., Bayat A. (2009). Exploring the Role of Stem Cells in Cutaneous Wound Healing. Exp. Dermatol..

[85] Guo S., DiPietro L.A. (2010). Factors Affecting Wound Healing. J. Dent. Res..

[86] Purba T.S., Haslam I.S., Poblet E., Jiménez F., Gandarillas A., Izeta A., Paus R. (2014). Human Epithelial Hair Follicle Stem Cells and Their Progeny: Current State of Knowledge, the Widening Gap in Translational Research and Future Challenges: Prospects & Overviews. BioEssays.

[87] Mascré G., Dekoninck S., Drogat B., Youssef K.K., Brohée S., Sotiropoulou P.A., Simons B.D., Blanpain C. (2012). Distinct Contribution of Stem and Progenitor Cells to Epidermal Maintenance. Nature.

[88] Ito M., Liu Y., Yang Z., Nguyen J., Liang F., Morris R.J., Cotsarelis G. (2005). Stem Cells in the Hair Follicle Bulge Contribute to Wound Repair but Not to Homeostasis of the Epidermis. Nat. Med..

[89] Langton A.K., Herrick S.E., Headon D.J. (2008). An Extended Epidermal Response Heals Cutaneous Wounds in the Absence of a Hair Follicle Stem Cell Contribution. J. Investig. Dermatol..

[90] Kulshreshtha G., Rathgeber B., Stratton G., Thomas N., Evans F., Critchley A., Hafting J., Prithiviraj B. (2014). Feed Supplementation with Red Seaweeds, Chondrus Crispus and Sarcodiotheca Gaudichaudii, Affects Performance, Egg Quality, and Gut Microbiota of Layer Hens. Poult. Sci..

[91] Walter M.N.M., Wright K.T., Fuller H.R., MacNeil S., Johnson W.E.B. (2010). Mesenchymal Stem Cell-Conditioned Medium Accelerates Skin Wound Healing: An in Vitro Study of Fibroblast and Keratinocyte Scratch Assays. Exp. Cell Res..

[92] Coulombe P.A. (2003). Wound Epithelialization: Accelerationg the Pace of Discovery. J. Investig. Dermatol..

[93] Leane M.M., Nankervis R., Smith A., Illum L. (2004). Use of the Ninhydrin Assay to Measure the Release of Chitosan from Oral Solid Dosage Forms. Int. J. Pharm..

[94] Prochazkova S., Vårum K.M., Ostgaard K. (1999). Quantitative Determination of Chitosans by Ninhydrin. Carbohydr. Polym..

[95] Hudzicki J. (2009). Kirby-Bauer Disk Diffusion Susceptibility Test Protocol. https://asm.org/getattachment/2594ce26-bd44-47f6-8287-0657aa9185ad/Kirby-Bauer-Disk-Diffusion-Susceptibility-Test-Protocol-pdf.

